# The 5-Year Onset and Regression of Diabetic Retinopathy in Chinese Type 2 Diabetes Patients

**DOI:** 10.1371/journal.pone.0113359

**Published:** 2014-11-17

**Authors:** Peiyao Jin, Jinjuan Peng, Haidong Zou, Weiwei Wang, Jiong Fu, Binjie Shen, Xuelin Bai, Xun Xu, Xi Zhang

**Affiliations:** 1 Department of Ophthalmology, Shanghai First People’s Hospital, Shanghai Jiao Tong University, Shanghai, 20080, China; 2 Department of Preventative Ophthalmology, Shanghai Eye Disease Prevention and Treatment Center, Shanghai, 200040, China; 3 Beixinjing Community Health Service Center, Shanghai, China; Massachusetts Eye & Ear Infirmary, Harvard Medical School, United States of America

## Abstract

**Purpose:**

To determine the rate and risk factors of diabetic retinopathy (DR) onset and regression in Chinese type 2 diabetes mellitus patients.

**Methods:**

This is a 5-year community-based prospective study. The demographic information, systemic examination results and ophthalmological test results of each participant were collected. The study outcomes were DR incidence, defined as the onset of DR in at least one eye, and DR regression, defined as full regression from existing DR to no retinopathy without invasive treatments. The associations between each potential risk factor and the outcomes were studied.

**Results:**

In total, 778 participants were enrolled. There were 322 patients without DR at baseline, of which 151 participants developed DR during follow-up (DR incidence rate = 46.89%). Baseline hyperglycemia and high blood pressure were two independent risk factors associated with DR incidence. Among the 456 participants with existing DR at entry, 110 fully recovered after 5 years (DR regression rate = 24.12%). Low baseline glucose and low serum triglyceride were two independent factors associated with DR regression.

**Conclusions:**

DR incidence occurred more frequently in patients with hyperglycemia and high blood pressure. DR regression occurred mostly in patients with lower glucose and lower serum triglyceride levels among Chinese type 2 diabetes patients.

## Introduction

Diabetic retinopathy (DR) is a common complication of diabetes mellitus. Although many treatments are available, DR remains the most common cause of blindness among people 30 to 69 years of age in several Western countries. [1], [2] It is universally believed that the key to controlling diabetic retinal complications is to prevent them from happening. To achieve this target, it is necessary to identify the factors associated with DR onset.

According to a recent Chinese population-based study [3], the age-standardized prevalence of diabetes in Chinese adults in 2010 was 9.7%, representing at least 92 million Chinese diabetes patients, which might be greater than that found in any other country. Most of these patients are suffering from type 2 diabetes. However, many classic DR incidence studies have been conducted among type 1 diabetes patients [4], [5], while studies targeting type 2 diabetes patients are relatively fewer. Moreover, a previous study suggested that large differences may exist in DR onset among various populations [6]. Most former studies were undertaken in Caucasian populations [4], [7]–[9], whereas data regarding the incidence of DR in Asian races, especially in the Chinese population, are scarce. To the best of our knowledge, only one with a Chinese population has been conducted to date in a hospital setting in Hong Kong and found a 20.3% 4-year cumulative DR incidence in a total of 212 type 2 diabetes patients [10]. Thus, a community-based prospective study with a larger population will provide more evidence to reveal the risk factors of DR incidence in Chinese type 2 diabetes patients.

Since 2003, our study group has been helping diabetes residents in the Beixinjing community to prevent and control DR progression through glucose control [11]. During our work, we have witnessed fully regressed retinopathy in several DR patients. The interesting part was that the regression happened without invasive treatments such as photocoagulation, intraocular medicine injection or surgery. These patients only accepted systematic treatments to improve microcirculation and to control high glucose levels, hypertension and hyperlipidemia. Although many studies have discussed the effect of photocoagulation or intravitreal bevacizumab (Avastin) treatments in DR regression [12]–[14], studies concerning DR regression without invasive treatments are limited. A few observational studies [15], [16] and case reports [17], [18] have reported the phenomenon of DR regression without discussing the factors associated with it. A famous randomized controlled trial (RCT) reported the influence of Candesartan in DR regression in type 2 diabetes patients [19]. Although the reliability of observational studies is not as good as RCTs in determining the effect of a treatment, observational studies can obtain the DR regression rate in a community population. As far as we know, no observational population study has reported the systemic factors associated with DR regression in a community population before. Although late stage DR can be contained by photocoagulation, intraocular medicine or vitreoretinal surgery, it is often at the expense of functional retina and visual performance and often results in poor prognoses [20], [21]. By studying the factors associated with DR regression, we can provide early stage DR patients with more options to not only slow the progression of retinopathy but also to reverse the existing disease, thus avoiding late-stage retinopathy and achieving better prognoses.

The results we report here were based on a 5-year prospective study of 778 participants in a community-setting cohort in Shanghai, China. The aim of this study was to determine the onset and regression rate of DR and the associated factors in Chinese type 2 diabetes patients.

## Methods

### Setting and participants

The Beixinjing community is located in the southwestern corner of the Shanghai urban district. The population of this community has generally remained stable over the past 20 years. In 1995, a health information database including almost all of the residents was created and has been updated annually by the Beixinjing Community Health Service Center [11].

During follow-up, participants took necessary medicines to control systemic diseases. For example, individualized anti-hyperglycemia therapy, including metformin, sulfonylureas, meglitinides, glitazones and insulin injections, or behavioral therapy, such as exercise, diet control, and smoking cessation, were used to help control hyperglycemia. Patients’ blood glucose levels were monitored by capillary blood glucose determination once a week, and their hemoglobin A1c (HbA1c) levels were measured once per year. Participants who also had high blood pressure continued their antihypertensive therapy during follow-up using common drugs including diuretics, β-adrenoceptor blocking drugs, angiotensin-converting enzyme inhibitor (ACEI), angiotensin II receptor antagonist (ARB), and calcium channel blocking drugs. Participants with hyperlipemia or hypercholesterolemia took drugs such as statins to lower their lipid levels. Many patients also used calcium dobesilate drugs to improve microcirculation.

As the clinical profile of DR suggests [22], most DR patients in this community with severe non-proliferative DR or proliferative retinopathy accepted photocoagulation, intraocular medicine injection or surgery to control DR progression. Because those treatments would influence the outcome of our study, patients who accepted invasive treatment before or during the study period were excluded. Only those patients without obvious DR, mild non-proliferative DR or moderate non-proliferative DR and those with severe DR but who refused to undertake invasive treatments were included.

The inclusion criteria for the present study were as follows: patients with diagnosed type 2 diabetes according to the WHO definition [23], more than 18 years of age, no previous eye surgery, no history of photocoagulation or intraocular medicine, willingness to accept a general physical examination, and willingness to sign the informed consent form. The exclusion criteria were as follows: suffering from severe systemic disease other than diabetes (such as severe heart/brain vessel disease and cancer), suffering from severe eye diseases other than DR (such as severe cornea opacity, severe cataracts, glaucoma, or retinal detachment), and accepting eye surgery, intraocular injection or photocoagulation as DR treatment during follow-up.

This study was conducted according to the tenets of the Declaration of Helsinki and was approved by the Institutional Review Board of the Shanghai First People's Hospital, Shanghai Jiao Tong University. All of the participants understood the study protocol and provided written informed consent to take part in this study.

### Anthropometric and Laboratory Measurements

The baseline survey was conducted in 2007. We acquired some of the participants’ information from the community health information database, including age, gender, diabetes duration, diabetes onset age, occupation, education level, and general and ophthalmological medical history. The diabetes duration was the interval between the diagnosis of diabetes and the baseline examination. The patients’ heights, weights, and systolic and diastolic blood pressures were measured by trained doctors. The blood pressure was measured in a supine position on the right arm using a standard mercury sphygmomanometer. Hypertension was defined as a systolic blood pressure value ≥140 mmHg and/or a diastolic blood pressure value ≥90 mmHg. BMI was calculated as weight (kg)/height (m)^2^. According to the guidelines released by the Chinese Ministry of Health, the definition of obesity was BMI≥28 [24]. Laboratory tests included measurements of serum triglycerides, total cholesterol, serum creatinine, urinary microalbumin and HbA1c. Serum creatinine, total cholesterol and triglycerides were measured using an enzymatic assay. Urinary microalbumin was analyzed by nephelometry. HbA1c was measured by ion exchange chromatography. Serum triglyceride levels ≥1.7 mmol*L^−1^ were considered hyperlipidemia, and serum total cholesterol levels ≥5.2 mmol*L^−1^ were considered hypercholesterolemia. Microalbuminuria levels ≥30 mg/L and serum creatinine levels ≥104 umol*L^−1^ suggested renal dysfunction. During follow-up, the HbA1c value of each participant was measured once per year, and the average HbA1c of the five years was recorded. All control values were consistent with the standards recommended by the Shanghai Clinical Testing Center.

### General Eye Examinations and Retinopathy Assessments

Eye examinations were conducted during the baseline survey (2007) and the final survey (2012). Visual acuity was tested by EDTRS tables with a 300-lux light. The eyelid, conjunctiva, cornea, iris, and lens were examined with a slit-lamp microscope (YZ-5, Liuliu Medical Instrument Company, Suzhou, China). All of the participants were screened for DR using the standardized protocol described in our previous paper [25]. The procedure was generally as follows. First, the patient’s vitreous and fundus were examined by a direct ophthalmoscope, and the posterior pole was then photographed with a non-mydriatic funduscopic camera (CR-DGI, Canon, Tokyo, Japan). Two 45-degree digital retinographs were obtained per eye. The retinographs were taken using the technique described in the EURODIAB study [26], with one centering on the macula and the other nasal to the optic disk. Once DR was suspected by our screening exams, the patient received a series of further exams in our hospital, including optical coherence tomography and fundus fluorescein angiography, to confirm the diagnosis and severity. Two trained ophthalmological doctors independently classified the DR grades according to the well-accepted “International Clinical Diabetic Retinopathy and Diabetic Macular Edema Disease Severity Scale” [27]. This system has five DR grades: no obvious DR (grade 0), mild non-proliferative DR (grade 1), moderate non-proliferative DR (grade 2), severe non-proliferative DR (grade 3), and proliferative retinopathy (grade 4). The two assessors were masked during the reading procedure. A sample of retinal photographs was graded again to assess the validity. Overall, there was a high degree of agreement on the assessment of retinopathy with respect to internal and inter-observer reliability (k1 = 0.874 and k2 = 0.869). Any unresolved disagreements between the two assessors were referred to the group leader (H.Z.) for arbitration.

There were two main outcomes assessed in the present study: DR incidence, defined as the onset of DR from grade 0 to grade 1–4 in at least one eye, and DR regression, defined as the decrease of DR severity from grade 1–4 to grade 0 in at least one eye and no DR worsening in the other eye.

### Statistical Analysis

The data were independently input by two staff members into an Access database. SAS (version 9.3 SAS Institute, Cary, NC, USA) was used for all statistical analyses. The baseline characteristics are presented as the means ± standard deviation for continuous variables and as rates (proportions) for categorical data. The data distribution was examined using the Kolmogorov-Smirnov Test. For normally distributed continuous variables, t tests were used to compare the means between groups. For variables that did not follow Gaussian distribution, a Kruskal-Wallis H test was used. The categorical variables were compared with the Chi-square test. From the univariate logistic analyses, variables with p values under 0.5 were considered for entry into multiple logistic regression analysis. Stepwise multiple logistic regression analysis was used to determine whether potential risk factors (including age, gender, diabetes duration, diabetes onset age, occupation, education level, BMI, baseline HbA1c, average HbA1c, high blood pressure, serum triglycerides, total cholesterol, serum creatinine and urinary microalbumin) were associated with DR onset and regression (inclusion value = 0.05 and exclusion value = 0.05). Statistical significance was defined as *p*<0.05 (two tailed).

## Results

In 2007, a total of 811 diabetes patients were enrolled in our study, of which 778 completed the whole study. Among the 33 excluded patients, 6 had refractive media opacity that affected the quality of their retinal photographs, and 27 patients, including 15 patients with grade 4 DR and 12 patients with grade 3 DR, accepted surgery or photocoagulation during follow-up. A total of 623 of the included diabetes patients were also suffering from high blood pressure; and most of these patients took medicine such as angiotensin converting enzyme inhibitors (ACEI), angiotensin receptor blockers (ARB) and traditional Chinese medicine to control their hypertension. However, most of them could not provide us with the exact dosage of their drugs. A total of 322 participants did not have DR in either eye at entry and were studied for DR incidence. The remaining 456 participants had existing DR in at least one eye at entry, and they were studied for DR regression. No missing cases were found during the five-year follow-up period.

### Risk factors for the onset of DR

At the time of the baseline survey in 2007, there were 322 DR-free participants, of which 142 (44.10%) were male. The youngest participant was 19 years of age, and the oldest was 88 years of age. A total of 85.09% of the participants were more than 60 years old. In 6 participants, diabetes had been diagnosed within the last year, and the longest duration of diabetes was 30 years. A total of 50.93% of the patients had diabetes for more than ten years. All participants’ microalbuminuria values were less than 30 mg/L. Detailed information of all of the baseline DR-free participants are summarized in Table 1.

**Table 1:** Characteristics of baseline DR-free patients and comparison of the clinical characteristics between patients with DR development during follow-up and patients without DR development.

	Total	DR development	No DR development	Statistical value	P value
Number of participants	322	151 (46.89)	171 (53.11)		
Age (years)	66.07±13.18	67.47±13.38	64.84±12.90	2.52*	0.01
age<60	48 (14.91)	20 (13.25)	28 (16.37)	4.19^†^	0.04
60≤age<70	116 (36.02)	49 (32.45)	67 (39.18)		
70≤age<80	136 (42.23)	67 (44.37)	69 (40.35)		
age>80	22 (6.83)	15 (9.93)	7 (4.09)		
Male gender	142 (44.10)	64 (42.38)	78 (45.61)	0.34^†^	0.56
High school degree or above	205 (63.66)	96 (63.58)	109 (63.74)	<0.01^†^	0.98
Diabetes onset age (years)	55.13±14.90	56.72±15.25	53.72±14.48	2.08*	0.04
Diabetes duration (years)	10.95±7.89	10.75±7.89	11.12±7.90	−0.48*	0.63
duration<10	158 (49.07)	78 (51.66)	80 (46.78)	0.02^†^	0.90
10≤duration<20	102 (31.68)	41 (27.15)	61 (35.67)		
20≤duration<30	60 (18.63)	31 (20.53)	29 (16.96)		
duration≥30	2 (0.62)	1 (0.66)	1 (0.58)		
Obesity	37 (11.49)	20 (13.25)	17 (9.94)	0.86^†^	0.35
High blood pressure	129 (40.06)	70 (46.36)	59 (34.50)	4.69^†^	0.03
High serum creatinine	12 (3.73)	7 (4.64)	5 (2.92)	0.65^†^	0.42
Hyperlipidemia	114 (35.40)	55 (36.42)	59 (34.50)	0.13^†^	0.72
Hypercholesterolemia	202 (62.73)	93 (61.59)	109 (63.74)	0.16^†^	0.69
Baseline HbA1c (%)	7.42±2.19	7.65±2.09	7.22±2.27	2.52*	0.01
Average HbA1c (%)	6.84±1.43	6.96±1.48	6.74±1.38	2.02*	0.04

Note: *Wilcoxon two-sample test ^†^Chi-Square test.

After five years, 151 patients developed DR in at least one eye (DR incidence rate = 46.89%), including 87 patients (57.62%) with grade 1 DR, 36 (23.84%) with grade 2 DR, 21 (13.91%) with grade 3 DR, and 7 (4.63%) with grade 4 DR. Among patients who developed DR, 94.04% (n = 142) developed DR in both eyes. The data indicated that patients with greater age, later diabetes onset, higher blood pressure, higher baseline glucose and higher average glucose were more likely to develop DR (Table 1). From the univariate logistic regression, participant age, age of diabetes onset, BMI, blood pressure, serum creatinine baseline HbA1c and average HbA1c were entered into the multivariate logistic analysis (p<0.5). Multivariate regression analysis suggested that both high blood pressure (OR = 1.80, 95% CI 1.14–2.86, *p* = 0.01) and high baseline HbA1c (OR = 1.12, 95% CI 1.01–1.24, *p* = 0.03) were independent factors associated with DR incidence.

### Factors related to DR regression

There were 456 participants with existing DR at entry, and 40.36% (n = 184) of them were male. Their ages ranged from 20 years of age to 90 years of age, and 84.65% were more than 60 years of age. Their diabetes duration ranged from 1 year to 33 years, and 43.42% of them had diabetes for more than ten years. All participants had microalbuminuria values less than 30 mg/L. Detailed information of the baseline characteristics of the participants with pre-existing DR is summarized in Table 2.

**Table 2:** Characteristics of patients with DR at baseline and comparison of the clinical characteristics between patients with DR regression during follow-up and patients without DR regression.

	Total	DR regression	No DR regression	Statistical value	P value
Number of participants	456	110	346		
Age (years)	68.76±12.27	67.15±13.72	69.27±11.75	−1.39*	0.16
age<60	52 (11.40)	18 (16.36)	34 (9.83)	1.44^†^	0.23
60≤age<70	135 (29.61)	28 (25.45)	107 (30.92)		
70≤age<80	211 (46.27)	53 (48.18)	158 (45.66)		
age>80	58 (12.72)	11 (10.00)	47 (13.58)		
Male gender	184 (40.35)	43 (39.09)	141 (40.75)	0.10^†^	0.76
High school degree or above	261 (57.24)	64 (58.18)	197 (56.94)	0.05^†^	0.81
Diabetes onset age (years)	56.98±13.51	56.88±15.02	57.01±13.01	0.66*	0.51
Diabetes duration (years)	11.78±8.01	10.26±7.53	12.27±8.11	−2.55*	0.01
duration<10	208 (45.61)	61(55.45)	147 (42.49)	4.71^†^	0.03
10≤duration<20	149 (32.68)	30 (27.27)	119 (34.39)		
20≤duration<30	93 (20.39)	18 (16.36)	75 (21.68)		
duration≥30	6 (1.32)	1 (0.91)	5 (1.45)		
Obesity	80 (17.54)	20 (18.18)	60 (17.34)	0.04^†^	0.84
High blood pressure	194 (42.54)	46 (41.82)	148 (42.77)	0.03^†^	0.86
High serum creatinine	9 (1.97)	0	9 (2.60)	2.92^†^	0.09
Hyperlipidemia	182 (39.91)	35 (31.82)	147 (42.49)	3.96^†^	0.04
Hypercholesterolemia	292 (64.04)	67 (60.91)	225 (65.03)	0.62^†^	0.43
Baseline HbA1c (%)	7.20±2.25	5.66±1.76	7.68±2.18	−10.69*	<0.01
Average HbA1c (%)	6.73±1.41	5.99±0.87	7.05±1.48	−9.35*	<0.01

Note: *Wilcoxon two-sample test ^†^Chi-Square test.

One hundred and five patients exhibited DR regression in both eyes, and another 5 patients had DR regression in one eye and no DR worsening of the other eye. A total of 110 participants had their DR severity grade regressed to grade 0 in the five-year follow-up period (DR regression rate = 24.12%), including 80 patients from grade 1 DR (72.73%) and 30 patients from grade 2 DR (27.27%). No patients from grade 3 or grade 4 exhibited full regression to grade 0.

DR regression often occurred in patients with shorter diabetes duration, normal serum triglyceride levels, lower baseline HbA1c levels and lower average HbA1c levels (Table 2). From the univariate logistic regression, participant age, diabetes duration, serum creatinine, serum triglyceride, serum total cholesterol, baseline HbA1c and average HbA1c were entered into the multivariate logistic analysis (p<0.5). The multivariate regression analysis suggested that low baseline HbA1c (OR = 0.53, 95% CI 0.44–0.63, *p*<0.01) and low serum triglyceride levels (OR = 0.77, 95% CI 0.60–0.99, *p* = 0.04) were two independent factors related to DR regression.

## Discussion

To the best of our knowledge, the present study was not only the first prospective study of DR incidence in a community setting cohort of Chinese type 2 diabetes patients but also the first observational study aimed at exploring the systemic factors related to DR regression in a community population.

Previous studies have found several risk factors of DR incidence in type 2 diabetes patients, including hypertension, hyperglycemia, long diabetes duration, smoking, insulin therapy, and obesity. Our research suggested that high blood pressure and high glucose are two independent risk factors for DR in Chinese diabetes patients, which is consistent with the findings of previous research conducted in other populations [7], [9], [28]–[32]. The complex pathogenesis of hyperglycemia and hypertension in retinal damage remains unclear, but significant evidence has indicated that chronic hyperglycemia and hypertension lead to oxidative injury, microthrombi formation, cell adhesion molecule activation, leukostasis and cytokine activation including vascular endothelial growth factor, insulin-like growth factor-1, angiopoetin-1 and -2, stromal-derived factor-1, fibroblast growth factor-2 and tumor necrosis factor. The combination of these cytokines leads to further retinal damage [33]. Although diabetes duration is recognized as a risk factor associated with diabetes retinopathy in Caucasian populations, there is no evidence in the present study that could independently associate diabetes duration with DR onset. This discrepancy might result from the inaccuracy of diabetes duration determinations. Because the onset of type 2 diabetes is often unknown, the disease could exist long before the patient is diagnosed.

Although DR incidence of type 2 diabetes patients has been reported in several countries [5], [7], [9], [28]–[30], [34], there is a large difference between the incidence values, which vary from 3.9% (for 5 years) [34] to 47.5% (for 4 years) [30]. The 5-year cumulative incidence of DR presented in our study was 46.89%, which is similar to that found in a study conducted in Iran [30]. Former studies have suggested that the Asian population is more sensitive to high glucose than Caucasian patients and is thus more likely to develop DR [35], [36]. The racial difference might explain the higher incidence rate reported in Iran and in our study compared with several studies conducted in Western countries. Moreover, although diabetes duration is not an independent factor associated with DR onset in our study, the mean diabetes duration of our participants is similar to that of the participants in the Iranian research study and is much longer than that found in other studies conducted in Asian populations [10], [31]. This might contribute to the high incidence rate.

Despite the high incidence rate, DR regression cases were quite common in our study, especially in patients with well-controlled glucose and mild baseline DR. Spontaneous diabetic retinopathy reversal has been noted by many ophthalmologists and reported by some researchers [15]–[18]; however, few have discussed its association with systemic factors. Compared with the 4.9% regression rate found in the Netherlands [15] and the 6.6% regression rate found in Hong Kong [10], the 24.12% DR regression rate in the present research is very high. This difference could be explained by the better systemic condition of our participants, which suggests that they had less severe diabetes complications. The inclusion criteria of our study ensured that the participants had no severe heart/brain vessel diseases, whereas the other two studies did not exclude patients with severe circulation problems. Furthermore, no patients in our study had abnormal microalbuminuria, whereas 24.4% of the participants in the Hong Kong study had albuminuria or proteinuria. The mean baseline HbA1c of our participants was 7.20%±2.25% (55±24.6 mmol/mol), which is significantly lower than the mean levels of 8.1%±1.5% (65±16.4 mmol/mol) and 7.8%±1.7% (62±18.6 mmol/mol) in the other two studies. Because our results indicated that the maintenance of euglycemia is associated with DR regression, the strict glucose target could lead to less worsening and further regression of retinopathy. Another possible explanation of the high regression rate found in the present study is that the drugs our patients used to control hypertension and to improve microcirculation may also promote DR regression. Previous studies have suggested that ACEI therapy has an additional protective effect in promoting the regression of DR [19], [37], [38], and it has been reported that pycnoyenol prevents progression of retinopathy and partly recovers visual acuity [39], [40]. Although there were previous case reports about PDR reversal [17], full reversal to grade 0 was not observed in late-stage DR patients in our study, which is consistent with a former RCT study about the effect of candesartan in patients with DR [19]. The study also reported that DR regression only occurred in participants with early retinopathy. Another possible reason is the lack of subjects because 15 PDR patients were excluded from the study for receiving invasive treatments during the follow-up, and only 2 PDR patients completed the study.

Our results suggested that if a DR patient’s HbA1c was decreased by 1% (10.9 mmol/mol), the chance of a full regression would be almost doubled. It has been reported that background retinopathy is not merely a process of cumulative increase in the number of microaneurysms but also reflects a dynamic balance between microaneurysm formation and disappearance. In patients with good glucose control, microaneurysm formation showed a decreasing trend, but the disappearance rate was not directly connected with glucose control [41], [42]. We speculated that this phenomenon could explain the reversal in our patients. Long-term maintenance of euglycemia leads to a lower rate of new microaneurysm formation, and the existing background microaneurysms eventually disappeared completely.

According to our results, low serum triglyceride is another systemic factor that is independently related to DR regression, which is in accordance with previous studies. Several studies have reported that serum lipid levels are associated with the severity of hard exudates [43]. Moreover, strict lipid-lowering therapy was found to be associated with hard exudate regression in clinically significant macular edema [44], and fibrate drugs have been reported to be able to reduce the need for photocoagulation in DR patients [43]. A review of previous research studies showed that animal experiments suggest that the reversibility of retinal flow abnormalities, which is the earliest change of DR damage, can only be observed in short disease-duration subjects [45], [46]. However, although the mean diabetes duration of the regression patients in our study was significantly shorter than those who did not regress, there was no evidence to independently relate diabetes disease duration to DR regression.

Our participants paid more attention to controlling glucose after the enrollment of the study, a large part of them had a decrease in HbA1c level during the follow-up period. Results showed that baseline HbA1c, instead of 5-year average HbA1c, are related to DR development and DR regression. We speculated that this might result from the “metabolic memory” phenomenon. In 1990, Roy and his colleagues coined the term “metabolic memory” to describe the hypothesis that systemic metabolic imbalance may continue to develop in patients who no longer have hyperglycemia [47]. Two well-known studies have proved this negative metabolic memory in Western populations, “The Diabetes Control and Complications Trial/Epidemiology of Diabetes Interventions and Complications Research Group” (DCCT) study in type 1 diabetes [48] and the “United Kingdom Prospective Diabetes Study” (UKPDS) in type 2 diabetes [49]. They found that among patients with similar glucose level, DR often occurred in those with higher initial HbA1c. We suspect that this might also apply to the positive metabolic memory of low glucose, which could explain the reason why DR reversal often occurred in patients with lower baseline HbA1c levels in our study.

Our research has some limitations. First, the study population was limited to one community and was not sufficiently large to represent all diabetes patients in Shanghai. Second, the retinographs were taken centering on only the macula and the optic disk; thus, retinopathy outside of these areas might have been missed. Third, this research only covered a 5-year period. The DR regression observed might be hard to maintain for a longer duration. Future longer-term, multicenter studies should be conducted to verify these results. In conclusion, DR incidence often occurred in patients with baseline hyperglycemia and high blood pressure. DR regression occurred mostly in patients with lower baseline glucose and lower serum triglyceride levels among Chinese type 2 diabetes patients.
